# Longitudinal Changes in Albuminuria and Estimated Glomerular Filtration Rate in Patients with Type 2 Diabetes Treated with Empagliflozin or Canagliflozin: A Real-World Cohort Study

**DOI:** 10.3390/jcm15218631

**Published:** 2026-11-05

**Authors:** Ching-Yao Cheng, Jaw-Horng Liou, Kao Tz Wei, Shang-Feng Tsai

**Affiliations:** 1 Department of Pharmacy, Taichung Veterans General Hospital, Taichung City 407, Taiwan; chingyao4937d@gmail.com (C.-Y.C.);; 2 School of Pharmacy, China Medical University, Taichung City 404, Taiwan; 3 Division of Nephrology, Department of Internal Medicine, Taichung Veterans General Hospital, Taichung City 407, Taiwan; 4 Department of Life Science, Tunghai University, Taichung City 407, Taiwan; 5 Department of Post-Baccalaureate Medicine, College of Medicine, National Chung Hsing University, Taichung City 402, Taiwan; 6 Division of Clinical Informatics, Department of Digital Medicine, Taichung Veterans General Hospital, 160, Sec. 3, Taiwan Boulevard, Taichung City 407, Taiwan

**Keywords:** empagliflozin, canagliflozin, diabetes mellitus, renal function, age, albuminuria, glomerular filtration rate

## Abstract

**Background:** Direct real-world comparisons of empagliflozin and canagliflozin regarding renal outcomes in patients with type 2 diabetes mellitus (T2DM) remain limited. This study compared longitudinal changes in the estimated glomerular filtration rate (eGFR) and urinary albumin-to-creatinine ratio (UACR). **Methods:** This retrospective observational cohort study included 215 adults with type 2 diabetes mellitus who received either empagliflozin (*n* = 115) or canagliflozin (*n* = 100) at a tertiary medical center. Renal outcomes, including estimated glomerular filtration rate (eGFR) and urine albumin-to-creatinine ratio (UACR), were evaluated over 18 months. Between-group differences in renal outcomes were assessed using multivariable regression models adjusted for relevant baseline clinical characteristics. Linear mixed-effects models were additionally employed to account for repeated measurements and evaluate differences in longitudinal trajectories between treatment groups. A reduction in UACR of ≥30% from baseline was evaluated as a clinically meaningful albuminuria response using multivariable logistic regression. Subgroup analyses were performed according to age and baseline UACR. **Results:** In multivariable linear regression analysis, the 18-month change in eGFR did not differ significantly between canagliflozin and empagliflozin (adjusted β, −0.43 mL/min/1.73 m^2^; 95% confidence interval [CI], −4.12 to 3.26; *p* = 0.819). Consistently, linear mixed-effects models showed no significant treatment-by-time interaction for eGFR (*p* for interaction = 0.356) or log-transformed UACR (*p* for interaction = 0.104). In the adjusted logistic regression analysis, canagliflozin was associated with lower odds of achieving a ≥30% reduction in UACR at 12 months compared with empagliflozin (adjusted odds ratio [OR], 0.40; 95% CI, 0.20–0.79; *p* = 0.008). However, this association was not statistically significant at 18 months (adjusted OR, 0.62; 95% CI, 0.32–1.18; *p* = 0.147). Subgroup interaction analyses did not demonstrate significant effect modification by age or baseline UACR (*p* for interaction = 0.859 and 0.626, respectively). **Conclusions:** In this real-world cohort of patients with type 2 diabetes mellitus, empagliflozin and canagliflozin showed comparable longitudinal eGFR and UACR trajectories over 18 months. Although a difference in the likelihood of achieving a ≥30% reduction in UACR was observed at 12 months, this finding was not sustained at 18 months and should be interpreted cautiously. Overall, our findings do not establish the superiority of either SGLT2 inhibitor in terms of renal outcomes. Further studies with larger populations and prospective designs are warranted.

## 1. Introduction

Diabetes mellitus (DM) is the predominant etiology of end-stage kidney disease (ESKD) globally. In the United States, DM accounts for 30% to 50% of ESKD cases [1]. Patients undergoing dialysis due to DM-associated ESKD exhibit significantly poorer survival outcomes compared to those with ESKD from non-DM-related causes. A recent study reported that DM with early kidney involvement reduces life expectancy by approximately 16 years [1]. The first-line pharmacologic intervention for diabetic kidney disease (DKD) is renin–angiotensin system inhibition (RASi) [2]. Subsequently, sodium–glucose cotransporter 2 inhibitors (SGLT2is) are introduced for glycemic control and DKD management, with strong supporting evidence in the Kidney Disease: Improving Global Outcomes (KDIGO) guidelines [2]. Sodium–glucose cotransporter 2 inhibitors (SGLT2is) have been shown to improve pre-specified renal outcomes, including reductions in albuminuria and preservation of glomerular filtration rate (GFR), in multiple cardiovascular outcome trials (CVOTs), such as EMPA-REG (Empagliflozin, Cardiovascular Outcomes, and Mortality in Type 2 Diabetes trial) (empagliflozin) [3], CANVAS (CANagliflozin cardioVascular Assessment Study) (canagliflozin) [4,5], and DECLARE-TIMI58 (Dapagliflozin Effect on Cardiovascular Events–Thrombolysis in Myocardial Infarction 58) (dapagliflozin) [6]. Furthermore, dedicated renal outcome trials, including CREDENCE [7,7], DAPA-CKD [8], and EMPA-KIDNEY [9], have provided additional evidence supporting the renoprotective benefits of SGLT2i in patients with DKD.

In previous studies, most participants had a high risk of cardiovascular disease (CVD) or pre-existing severe albuminuria. For individuals without significant CV factors or with low albuminuria, evidence remains limited. For canagliflozin, a post hoc analysis of the CANVAS program demonstrated that canagliflozin reduced albuminuria across all baseline albuminuria categories [10]. However, the magnitude of albuminuria reduction was greater in individuals with moderately and severely increased albuminuria, with significant heterogeneity among subgroups (*p* for heterogeneity < 0.001). The inclusion criteria for the CANVAS and CANVAS-R trials required participants to have type 2 DM with a high risk of CVD. Eligible participants were either ≥30 years old with a history of symptomatic atherosclerotic CVD or ≥50 years old with at least two additional risk factors, including diabetes duration > 10 years, systolic blood pressure > 140 mmHg despite antihypertensive therapy, current smoking, albuminuria, or high-density lipoprotein cholesterol < 38.7 mg/dL. As for empagliflozin, a secondary analysis [3] from the EMPA-KIDNEY trial demonstrated its efficacy in reducing the urinary albumin-to-creatinine ratio (UACR) in patients with baseline UACR levels below 200 mg/g. Additionally, the EMPA-REG OUTCOME trial [11] reported an 18% reduction in UACR at week 12 compared with a placebo and an increased likelihood of achieving a >30% reduction in UACR. However, EMPA-REG OUTCOME exclusively included patients with established CVD, limiting its generalizability to broader patient populations.

Therefore, this study aimed to compare longitudinal changes in UACR and eGFR between patients with type 2 diabetes treated with empagliflozin or canagliflozin who had no documented history of heart failure or ischemic heart disease. Additionally, we seek to evaluate potential differences in the efficacy of SGLT2 inhibitors across varying baseline urinary UACR levels.

## 2. Materials and Methods

### 2.1. Study Design and Population

This retrospective observational cohort study was conducted at the outpatient department of Taichung Veterans General Hospital, Taiwan. We identified adults with type 2 diabetes mellitus and diabetic kidney disease who received empagliflozin or canagliflozin between 1 May 2018, and 30 August 2021. The date of the first prescription of the study drug during the study period was defined as the index date. Eligible patients were aged 20 years or older and had at least 84 days of recorded use of the prescribed sodium–glucose cotransporter 2 inhibitor (SGLT2i). Patients who switched to another SGLT2i during the study period and those with a documented history of heart failure or ischemic heart disease were excluded. Dapagliflozin was not included because its use at our institution was relatively limited during the study period (Supplementary Figure S1).

The study was approved by the Institutional Review Board of Taichung Veterans General Hospital (approval no. CE25114A; approval date: 7 March 2025). The requirement for informed consent was waived because the analysis used de-identified clinical data.

### 2.2. Data Collection and Baseline Characteristics

Two pharmacologists independently reviewed the medical records and extracted demographic characteristics, clinical diagnoses, laboratory measurements, and medication records. Variables collected included age, sex, body mass index, hypertension, chronic kidney disease (CKD) stage, glycated hemoglobin (HbA1c), serum creatinine, urinary albumin-to-creatinine ratio (UACR), estimated glomerular filtration rate (eGFR), statin use, and renin–angiotensin system inhibitor use. eGFR was calculated using the Modification of Diet in Renal Disease equation. Baseline laboratory values were defined as measurements obtained within 90 days before the index date. When multiple measurements were available during this period, the measurement closest to the index date was selected as the baseline value. Laboratory measurements were obtained from routine clinical records. Serum creatinine and eGFR were monitored more routinely than UACR during follow-up; therefore, the availability of laboratory measurements varied across patients and time points. The number of patients included in each analysis depended on the availability of the required laboratory measurements.

Canagliflozin was prescribed at 100 mg once daily in all patients receiving this agent. Empagliflozin dosing followed institutional prescribing practices during the study period, with a dose of 25 mg daily for patients with an eGFR > 45 mL/min/1.73 m^2^ and 10 mg daily for those with an eGFR < 45 mL/min/1.73 m^2^. GLP-1 receptor agonists and non-steroidal mineralocorticoid receptor antagonists were not used at our institution during the study period.

### 2.3. Outcomes and Follow-Up

The outcomes were longitudinal changes in UACR and eGFR after initiation of empagliflozin or canagliflozin. Measurements were evaluated at baseline and at 3, 6, 9, 12, 15, and 18 months, as available in the medical records; UACR was summarized at baseline and at 3, 6, 12, 15, and 18 months in the current analyses. For each scheduled follow-up time point, a 60-day assessment window was defined, extending 30 days before and 30 days after the target date. When multiple laboratory measurements were available within a follow-up window, the measurement closest to the target date was selected. Changes from baseline were calculated only for patients with both an available baseline measurement and the corresponding follow-up measurement. Patients without the required measurements at a specific time point were excluded from that time-point analysis, and missing values were not imputed. The number of patients included in each analysis was determined by the availability of paired measurements for the respective outcome and follow-up time point. Prespecified subgroup analyses stratified patients by age (<65 versus ≥65 years) and baseline UACR (<200 versus ≥200 mg/g). The UACR threshold was selected to align with the threshold utilized in both the EMPA-KIDNEY [9] and the DAPA-CKD trial [12].

To account for potential baseline differences between the empagliflozin and canagliflozin groups, additional multivariable regression analyses were performed. Multivariable linear regression was used to evaluate the change in eGFR from baseline to 18 months (ΔeGFR), calculated as the 18-month eGFR minus the baseline eGFR. The model was adjusted for treatment group, baseline eGFR, age, sex, baseline glycated hemoglobin (HbA1c), hypertension, and baseline use of angiotensin-converting enzyme inhibitors or angiotensin receptor blockers (ACEi/ARBs). Multivariable logistic regression was performed to evaluate albuminuria response at 12 and 18 months. An albuminuria response was defined as a reduction in UACR of at least 30% from baseline, corresponding to a follow-up UACR value ≤ 70% of the baseline value. Separate models were constructed for the 12- and 18-month assessments and adjusted for age, sex, baseline eGFR, baseline log-transformed UACR, baseline HbA1c, hypertension, baseline ACEi/ARB use, and statin use. Empagliflozin was used as the reference treatment group.

### 2.4. Statistical Analyses

Categorical variables were summarized as counts and percentages, while continuous variables were expressed as medians and interquartile ranges (IQRs). Between-group differences in baseline characteristics were assessed using the chi-square test for categorical variables and the Mann–Whitney U test for continuous variables. Longitudinal changes in eGFR and log-transformed UACR were evaluated using linear mixed-effects models with patient-specific random intercepts to account for within-subject correlations among repeated measurements. Treatment group, follow-up time, and the treatment-by-time interaction were included as fixed effects. Models were adjusted for relevant baseline covariates, including age, sex, baseline eGFR, HbA1c, hypertension, and baseline use of renin–angiotensin system inhibitors, as appropriate for each outcome. The treatment-by-time interaction was used to assess differences in longitudinal trajectories between the two treatment groups. Multivariable linear regression was additionally performed to estimate the adjusted between-group difference in eGFR change from baseline to 18 months. Regression coefficients (β) and 95% confidence intervals (CIs) were reported, with heteroscedasticity-consistent HC3 robust standard errors. Multivariable logistic regression was used to estimate adjusted odds ratios (ORs) and 95% CIs for achieving a ≥30% reduction in UACR at 12 and 18 months. Empagliflozin was designated as the reference group; therefore, an OR below 1 indicated lower odds of achieving the specified albuminuria response in the canagliflozin group.

Subgroup analyses were conducted according to age (<65 or ≥65 years) and baseline UACR (<200 or ≥200 mg/g). Formal interaction analyses were performed to evaluate whether longitudinal UACR trajectories differed according to age or baseline UACR category. The Friedman test with Dunn–Bonferroni-adjusted pairwise comparisons was retained for exploratory within-group comparisons, while between-group differences in changes from baseline at individual follow-up time points were assessed using the Mann–Whitney U test. The Holm procedure was applied to adjust for multiple comparisons in time-specific UACR analyses. Subgroup findings were considered exploratory and interpreted cautiously.

All statistical tests were two-sided, and a *p* value < 0.05 was considered statistically significant. Analyses were performed using SPSS version 12.0 (SPSS Inc., Chicago, IL, USA).

## 3. Results

### 3.1. Baseline Status of the Whole Cohort

A total of 215 patients were included in the analyses presented in Table 1: 115 received empagliflozin and 100 received canagliflozin. The groups did not differ significantly in age, sex, body mass index, CKD stage, glycated hemoglobin (HbA1c), baseline UACR, hypertension, statin use, or renin–angiotensin system inhibitor use (all *p* > 0.05). However, the canagliflozin group had a higher median serum creatinine value than the empagliflozin group (1.1 [IQR, 0.8–1.4] vs. 0.9 [IQR, 0.8–1.2] mg/dL; *p* = 0.016) and a lower median eGFR (65.0 [IQR, 47.9–85.7] vs. 72.5 [IQR, 56.3–93.2] mL/min/1.73 m^2^; *p* = 0.048). The median baseline UACR was 35.3 mg/g (IQR, 10.2–308.2) in the canagliflozin group and 26.0 mg/g (IQR, 8.5–127.3) in the empagliflozin group, with no significant between-group difference (*p* = 0.403). ACE inhibitor or ARB use was comparable between the two groups (54.0% in the canagliflozin group and 51.3% in the empagliflozin group).

### 3.2. Longitudinal UACR and eGFR Trends

For the longitudinal analysis of UACR (Table 2), complete measurements across all scheduled assessment time points were available for 93 patients in the empagliflozin group and 80 patients in the canagliflozin group. These numbers represent the patients included in the complete-case longitudinal analysis and should not be interpreted as the total number of patients with baseline UACR measurements. Within-group analyses demonstrated a statistically significant change in UACR over time in the empagliflozin group (Friedman test, *p* = 0.001), whereas the change did not reach statistical significance in the canagliflozin group (*p* = 0.449). In the empagliflozin group, median UACR decreased from 41.90 mg/g (IQR, 9.40–149.60) at baseline to 21.10 mg/g (IQR, 10.80–106.05) at 18 months. Dunn–Bonferroni post hoc comparisons identified significant differences between months 3 and 12 and between months 3 and 18. In the canagliflozin group, median UACR was 46.35 mg/g (IQR, 12.20–493.83) at baseline and 31.50 mg/g (IQR, 12.40–210.13) at 18 months, with no statistically significant overall longitudinal change.

For eGFR (Table 2), significant within-group longitudinal changes were observed in both treatment groups (Friedman test, *p* < 0.001 for each group). In the empagliflozin group, median eGFR was 72.45 mL/min/1.73 m^2^ (IQR, 56.26–93.17) at baseline and 70.14 mL/min/1.73 m^2^ (IQR, 50.35–88.35) at 18 months. Post hoc comparisons identified significant differences between baseline and months 15 and 18, as well as between months 3 and 18. In the canagliflozin group, median eGFR decreased from 64.99 mL/min/1.73 m^2^ (IQR, 47.87–85.66) at baseline to 63.32 mL/min/1.73 m^2^ (IQR, 41.48–81.08) at 18 months. Significant differences were identified between baseline and months 12, 15, and 18.

These findings describe longitudinal changes within each treatment group (Table 2). However, differences in statistical significance between the two groups do not necessarily indicate differences in treatment effects. Direct between-group comparisons (Table 3) and adjusted longitudinal analyses are therefore necessary to evaluate the comparative renal effects of empagliflozin and canagliflozin.

### 3.3. Between-Group Comparisons of Changes from Baseline

In the unadjusted between-group comparisons, no statistically significant differences in UACR changes from baseline were observed between the empagliflozin and canagliflozin groups at any follow-up time point (3, 6, 12, 15, or 18 months; all *p* ≥ 0.312). At 18 months, the median change in UACR was −1.60 mg/g (IQR, −49.20 to 6.40) in the empagliflozin group and 0.35 mg/g (IQR, −33.78 to 11.25) in the canagliflozin group (*p* = 0.312). Similarly, changes in eGFR did not differ significantly between the two groups at any follow-up time point (3, 6, 12, 15, or 18 months; all *p* ≥ 0.264). At 18 months, the median change in eGFR was −3.67 mL/min/1.73 m^2^ (IQR, −10.48 to 4.03) in the empagliflozin group and −4.09 mL/min/1.73 m^2^ (IQR, −10.10 to 2.15) in the canagliflozin group (*p* = 0.789). The between-group difference in HbA1c change from baseline to 18 months was also not statistically significant (*p* = 0.625). Consistent with these findings, the adjusted linear mixed-effects analyses showed no significant treatment-by-time interaction for either eGFR (*p* for interaction = 0.356) or log-transformed UACR (*p* for interaction = 0.104), suggesting no statistically significant differences in longitudinal renal outcome trajectories between the two treatment groups.

### 3.4. Subgroup Analyses by Age

**Figure 1:**
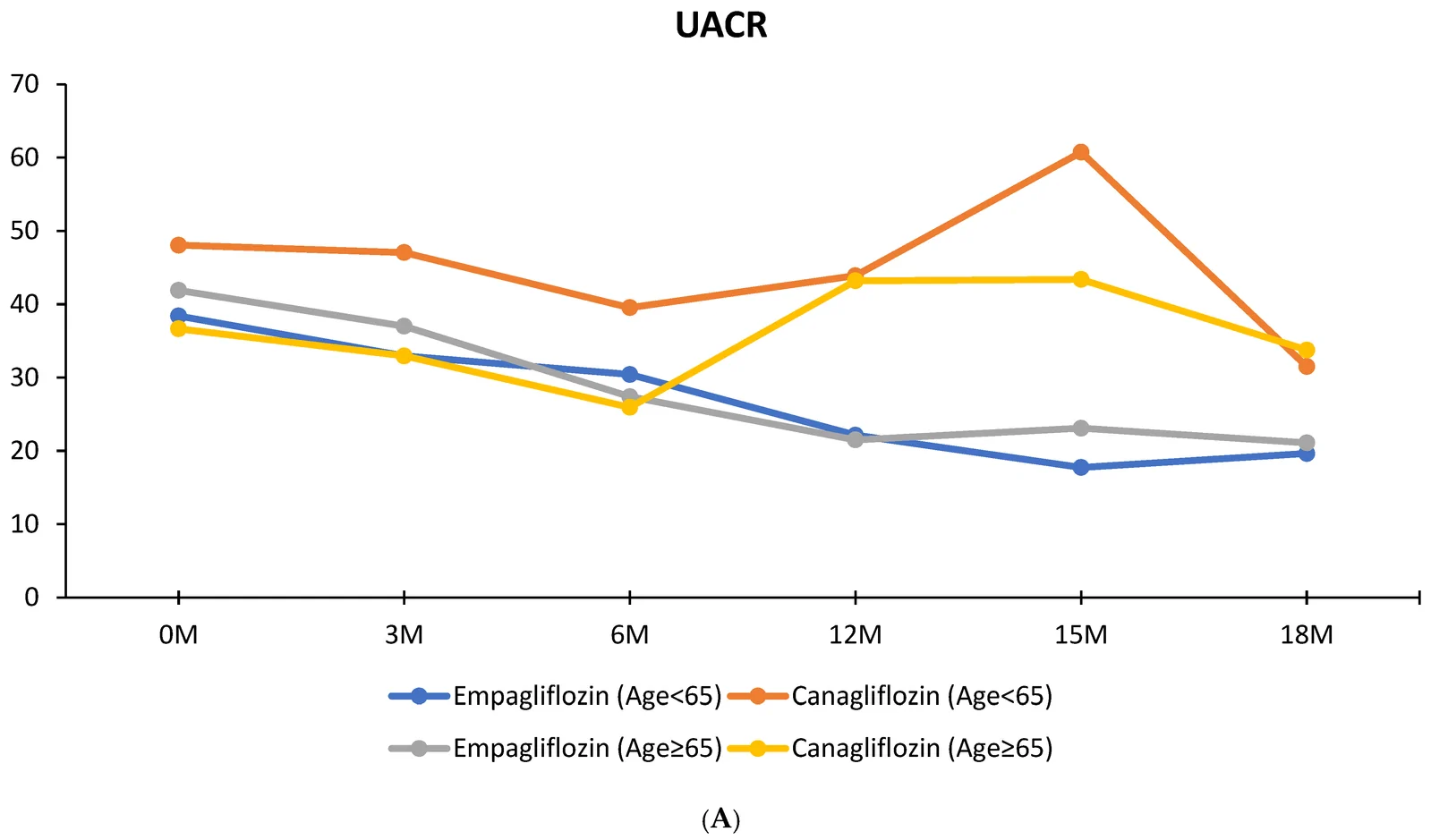

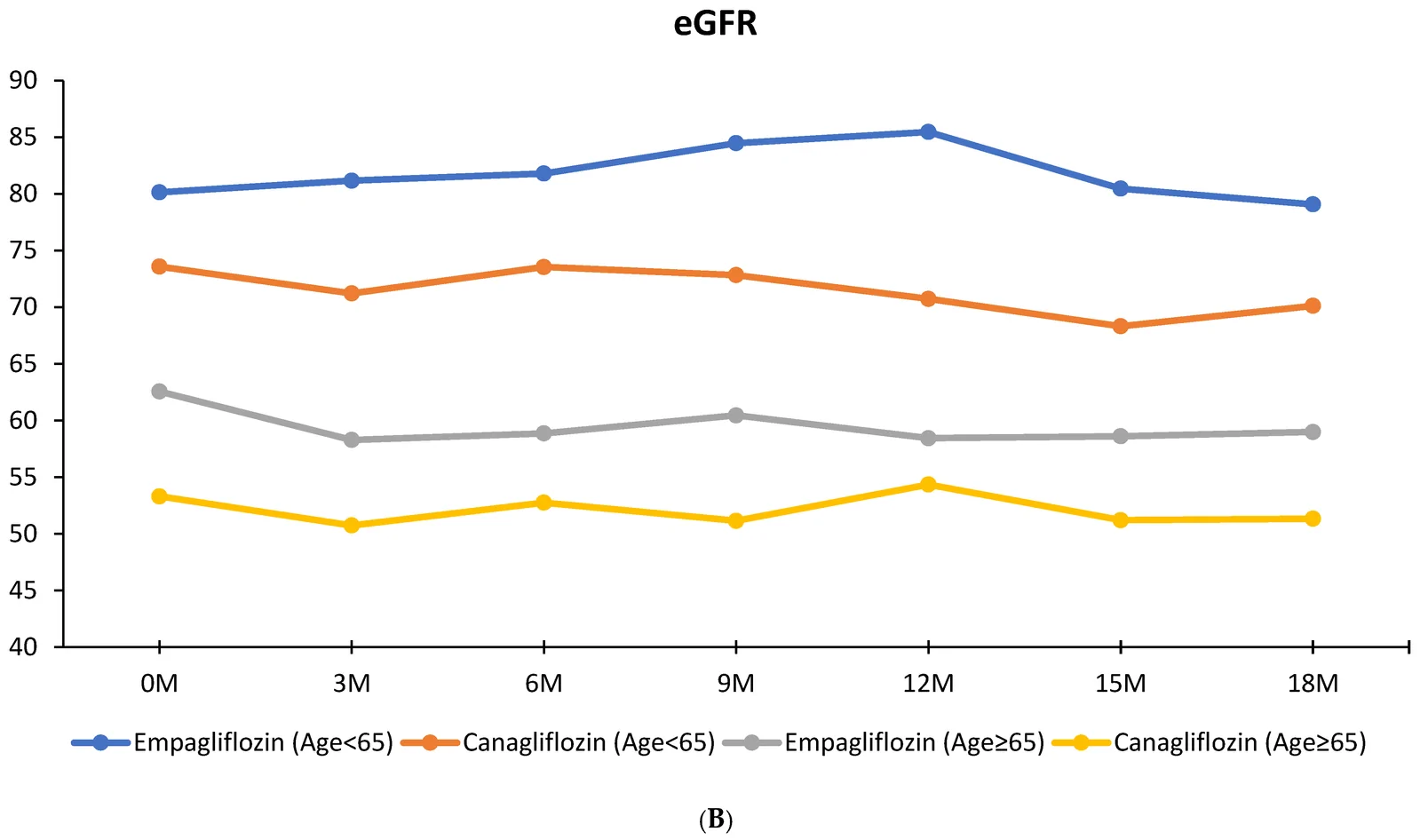
UACR and eGFR trends in empagliflozin and canagliflozin across different time periods in young and elderly groups. Figure 1 displays the median UACR and eGFR trajectories by age subgroup. The UACR curves show a downward pattern over the follow-up period among empagliflozin users in both age groups, whereas values among canagliflozin users fluctuate over time. The eGFR curves show different baseline levels by age and treatment group; the figure is descriptive and does not by itself establish a between-treatment difference.

The age-stratified longitudinal trajectories of UACR and eGFR are illustrated in Figure 1A and Figure 1B, respectively. In the age-stratified analyses (Supplementary Tables S1 and S2), within-group longitudinal changes in UACR were statistically significant among patients aged ≥65 years receiving empagliflozin (*p* = 0.004), but not among those aged <65 years (*p* = 0.163). Neither age subgroup in the canagliflozin group showed a statistically significant longitudinal change in UACR (*p* = 0.424 for age < 65 years and *p* = 0.957 for age ≥ 65 years). For eGFR, significant within-group longitudinal changes were observed among patients aged <65 years in both treatment groups (*p* = 0.003 for each group). Among patients aged ≥65 years, the within-group changes did not reach statistical significance (*p* = 0.054 for empagliflozin and *p* = 0.073 for canagliflozin). In the between-group comparisons of 18-month changes, no significant differences in UACR, eGFR, or HbA1c were observed among patients aged <65 years (*p* = 0.719, 0.463, and 0.811, respectively). Among patients aged ≥65 years, a nominal between-group difference was observed in UACR change, with a median change of −3.70 mg/g (IQR, −81.10 to 6.40) in the empagliflozin group and 2.20 mg/g (IQR, −17.78 to 23.13) in the canagliflozin group (*p* = 0.043). However, no significant between-group differences were observed in eGFR or HbA1c changes in this age subgroup (*p* = 0.647 and *p* = 0.635, respectively).

Furthermore, the treatment-by-time-by-age interaction analysis for longitudinal UACR did not demonstrate significant effect modification by age (*p* for interaction = 0.859). Therefore, the nominal between-group difference in UACR observed among patients aged ≥65 years should be interpreted cautiously and does not establish a differential treatment effect according to age.

### 3.5. Subgroup Analyses by Baseline UACR

Among patients with baseline UACR ≥200 mg/g, within-group longitudinal changes in UACR were statistically significant in both treatment groups (*p* = 0.003 for empagliflozin and *p* = 0.045 for canagliflozin) (Supplementary Tables S3 and S4). In contrast, no statistically significant longitudinal changes in UACR were observed in either treatment group among patients with baseline UACR < 200 mg/g (*p* = 0.097 for empagliflozin and *p* = 0.116 for canagliflozin). For eGFR, significant within-group longitudinal changes were observed in both treatment groups among patients with baseline UACR ≥ 200 mg/g (*p* < 0.001 for each group). Among patients with baseline UACR < 200 mg/g, the within-group eGFR change was statistically significant in the empagliflozin group (*p* = 0.040), but not in the canagliflozin group (*p* = 0.092).

In the between-group comparisons of 18-month changes, a nominal difference in UACR change was observed among patients with baseline UACR < 200 mg/g. The median change was −0.60 mg/g (IQR, −19.60 to 6.90) in the empagliflozin group and 1.70 mg/g (IQR, −3.30 to 11.10) in the canagliflozin group (*p* = 0.023). No significant between-group differences were observed in eGFR or HbA1c changes in this subgroup (*p* = 0.934 and *p* = 0.820, respectively). Among patients with baseline UACR ≥200 mg/g, no significant between-group differences were observed in UACR, eGFR, or HbA1c changes (*p* = 0.711, 0.946, and 0.137, respectively).

Furthermore, the treatment-by-time-by-baseline-UACR interaction analysis for longitudinal UACR did not demonstrate significant effect modification by baseline UACR category (*p* for interaction = 0.626). Therefore, the nominal between-group difference in UACR change observed among patients with baseline UACR < 200 mg/g should be interpreted cautiously and does not establish a differential treatment effect according to baseline albuminuria.

### 3.6. Additional Analyses of UACR Percentage Changes and Albuminuria Response

To further evaluate renal responses to SGLT2 inhibitor treatment, we performed additional analyses of percentage changes in UACR from baseline and the proportion of patients achieving a ≥30% reduction in UACR (Supplementary Tables S5 and S6). In the empagliflozin group, the median percentage change in UACR ranged from −15.3% at 6 months to −19.7% at 12 months, whereas the corresponding changes in the canagliflozin group ranged from 0.0% to −3.2% at these time points. However, no statistically significant between-group differences in percentage changes in UACR were observed at any follow-up time point (all *p* > 0.05). Regarding the albuminuria response, a ≥30% reduction in UACR was observed in 23.6%, 32.3%, 44.6%, 43.7%, and 39.3% of patients in the empagliflozin group at 3, 6, 12, 15, and 18 months, respectively. The corresponding proportions in the canagliflozin group were 17.8%, 28.7%, 26.1%, 33.0%, and 32.7%, respectively. In the unadjusted comparisons, a significantly higher proportion of patients receiving empagliflozin achieved a ≥30% reduction in UACR at 12 months compared with those receiving canagliflozin (44.6% vs. 26.1%, *p* = 0.010); no statistically significant differences were observed at the other follow-up time points. In the additional multivariable logistic regression analysis, after adjustment for age, sex, baseline eGFR, baseline log-transformed UACR, HbA1c, hypertension, ACE inhibitor/ARB use, and statin use, canagliflozin was associated with lower odds of achieving a ≥30% reduction in UACR at 12 months compared with empagliflozin (adjusted OR, 0.40; 95% CI, 0.20–0.79; *p* = 0.008). However, this association was not statistically significant at 18 months (adjusted OR, 0.62; 95% CI, 0.32–1.18; *p* = 0.147). Given that the difference was observed at only one follow-up time point among multiple comparisons, this finding should be interpreted cautiously as exploratory rather than definitive evidence of a difference in treatment efficacy.

### 3.7. Multivariable Analyses of Renal Outcomes

In the multivariable linear regression analysis, no significant difference in the 18-month change in eGFR was observed between the canagliflozin and empagliflozin groups after adjustment for baseline eGFR and other clinically relevant covariates (adjusted β, −0.43 mL/min/1.73 m^2^; 95% CI, −4.12 to 3.26; *p* = 0.819). In the multivariable logistic regression analysis, canagliflozin treatment was associated with lower odds of achieving a ≥30% reduction in UACR at 12 months compared with empagliflozin (adjusted OR, 0.40; 95% CI, 0.20–0.79; *p* = 0.008). However, this association was not statistically significant at 18 months (adjusted OR, 0.62; 95% CI, 0.32–1.18; *p* = 0.147) (Table 4).

Furthermore, in the adjusted linear mixed-effects models accounting for repeated measurements, the treatment-by-time interaction was not statistically significant for either eGFR (*p* for interaction = 0.356) or log-transformed UACR (*p* for interaction = 0.104). These findings suggest that the overall longitudinal trajectories of eGFR and UACR did not differ significantly between the two treatment groups.

## 4. Discussion

To our knowledge, this is the first study to compare the renal effects of empagliflozin and canagliflozin, including changes in UACR and eGFR, across different baseline age and albuminuria categories. Beyond their hemodynamic effects, SGLT2 inhibitors may exert renoprotective effects through anti-inflammatory and antifibrotic pathways. In this context, matrix metalloproteinases (MMPs) and their endogenous tissue inhibitors (TIMPs) play important roles in extracellular matrix remodeling and renal fibrosis, as recently reviewed by La Russa et al. [13]. Dysregulation of the MMP/TIMP balance may contribute to progressive renal fibrosis; however, whether these pathways are differentially modulated by empagliflozin and canagliflozin remains unclear. As MMPs, TIMPs, and fibrosis-related biomarkers were not evaluated in our study, their potential involvement in the observed renal outcomes warrants further investigation.

Regarding UACR, the complete-case longitudinal analysis demonstrated a significant within-group change over time in the empagliflozin group, whereas no statistically significant longitudinal change was observed in the canagliflozin group. However, direct between-group comparisons did not demonstrate significant differences in UACR changes at any follow-up time point, and the adjusted linear mixed-effects model showed no significant treatment-by-time interaction. In the subgroup analyses, nominal between-group differences in 18-month UACR changes were observed among patients aged ≥65 years and those with baseline UACR <200 mg/g. Nevertheless, the corresponding treatment-by-time-by-subgroup interaction analyses were not statistically significant. These findings therefore do not establish a differential treatment effect according to age or baseline albuminuria. Overall, the results do not demonstrate a consistent advantage of either agent in reducing albuminuria. The reduction in albuminuria with SGLT2i occurs through two key mechanisms: in the early stage of DKD, it is primarily driven by the restoration of TGF [14], which lowers intraglomerular pressure; in later stages, the effect is mediated by anti-inflammatory [15] and anti-fibrotic pathways [16]. The anti-fibrotic effects of empagliflozin and canagliflozin are similar, which explains why both medications can effectively reduce albuminuria in patients with higher baseline UACR. This also explains why both medications can reduce albuminuria in the elderly. Aging kidneys undergo progressive inflammation and fibrosis [17], similar to advanced diabetic kidney disease. Therefore, both empagliflozin and canagliflozin can lower albuminuria in individuals with higher baseline UACR or older age by reducing inflammation and fibrosis. On the contrary, in the early stage of DKD, albuminuria reduction mainly depends on lowering intraglomerular pressure, a process directly related to SGLT2 inhibition. Empagliflozin has much higher selectivity for SGLT2 over SGLT1 compared to canagliflozin [18]. This pharmacological difference may contribute to differences in their renal effects, including reductions in intraglomerular pressure and UACR, particularly in early DKD. In our study, although empagliflozin was associated with a higher proportion of patients achieving a ≥30% reduction in UACR at 12 months, this difference was not sustained at 18 months. Furthermore, no significant interaction was observed between treatment and baseline albuminuria category. Therefore, our findings do not establish a consistent advantage of empagliflozin across different baseline albuminuria levels.

Regarding eGFR, both empagliflozin and canagliflozin were associated with lower eGFR across nearly the entire cohort. The initial decline in eGFR with SGLT2i does not indicate renal injury but rather reflects tubuloglomerular feedback [14], leading to a reduction in intraglomerular pressure [14]. This mechanism contributes to a lower incidence of ESKD over long-term follow-up. This phenomenon has been well-documented in most studies investigating the effects of SGLT2i on eGFR [3,9,10,12]. The findings of our study indicate that the decrease in eGFR was more pronounced in individuals with higher baseline UACR for both medications. This is consistent with previous study [19]. In addition, our study demonstrated a more pronounced initial eGFR decline in the younger group, although elderly individuals also exhibited a trend toward lower eGFR. Several factors may explain this finding. First, younger patients, particularly those with diabetes or early-stage kidney disease, often have higher baseline GFR due to glomerular hyperfiltration [20]. Second, younger kidneys have greater functional reserve [21], allowing for a more rapid adjustment to changes in renal perfusion. In summary, if a young patient has significant albuminuria (high UACR), the initial eGFR dip tends to be even more pronounced.

Notably, canagliflozin did not result in a significant reduction in eGFR in individuals with lower baseline UACR. These within-group findings should be interpreted cautiously, as the difference in statistical significance does not necessarily indicate a difference in treatment effects between the two agents. Albuminuria reflects advanced glycation end product production and the accumulation of reactive oxidative species, which activate kinase cascades and increase pro-inflammatory cytokines and growth factors [22]. These processes contribute to structural changes in the kidney. In other words, lower baseline albuminuria indicates an earlier stage of (DKD). At this stage of DKD, the renal protective effects of SGLT2i primarily rely on reducing intraglomerular hyperfiltration. However, compared to empagliflozin (2500-fold), SGLT2 selectivity over SGLT1 is much lower (e.g., 250-fold) [18,23]. We speculate that differences in SGLT2 selectivity and the degree of SGLT1 inhibition may contribute to variations in the early hemodynamic response to these agents, including the initial eGFR dip. Nevertheless, our subgroup interaction analysis did not demonstrate a significant effect modification by baseline UACR. Therefore, this proposed mechanism remains speculative and requires further investigation.

This study has several limitations. First, its retrospective observational design limits the ability to establish causal relationships between SGLT2 inhibitor treatment and renal outcomes. Baseline renal function differed between the two treatment groups, with higher serum creatinine levels and lower eGFR in patients receiving canagliflozin. Furthermore, treatment allocation was not randomized, and the reasons for prescribing a specific SGLT2 inhibitor were not systematically documented. These differences raise the possibility of confounding by indication. Although multivariable regression and linear mixed-effects models were used to adjust for measured baseline characteristics and account for repeated measurements, residual confounding from unmeasured or incompletely captured factors cannot be excluded. Second, UACR and eGFR measurements were not available for all patients at every follow-up time point, resulting in varying sample sizes across analyses. In particular, UACR was not routinely measured at every clinical visit, which may have introduced selection bias if the availability of follow-up measurements was related to patients’ clinical conditions or treatment responses. Although linear mixed-effects models incorporated available repeated measurements, potential bias related to missing data cannot be completely ruled out. Third, detailed information on treatment adherence, dose modifications, treatment discontinuation, and longitudinal changes in concomitant medications was not consistently available. Although baseline HbA1c levels were comparable between the two groups, differences in concomitant oral antidiabetic therapies could not be systematically evaluated. In addition, GLP-1 receptor agonist use was not documented in this cohort during the study period; therefore, its potential influence on renal outcomes could not be assessed. Diabetes duration and longitudinal changes in renin–angiotensin system inhibitor therapy were also unavailable for comprehensive adjustment. These factors may have contributed to residual confounding in the observed renal outcomes. Fourth, multiple comparisons were performed across follow-up time points and clinical subgroups, increasing the possibility of chance findings. Although additional treatment-by-time-by-subgroup interaction analyses did not demonstrate significant effect modification by age or baseline UACR, the relatively small sample size may have limited the statistical power to detect subgroup differences. Accordingly, the subgroup findings and isolated time-point differences, particularly those involving albuminuria responses, should be interpreted as exploratory rather than confirmatory. Fifth, our analysis primarily focused on changes in eGFR and UACR rather than hard clinical outcomes, such as sustained eGFR decline, progression to end-stage kidney disease, initiation of kidney replacement therapy, or mortality. Therefore, the comparative long-term clinical benefits of empagliflozin and canagliflozin could not be determined. Furthermore, patients with documented heart failure or ischemic heart disease were excluded, which may limit the generalizability of our findings to the broader population with type 2 diabetes and established cardiovascular disease. Finally, we did not measure renal fibrosis, inflammatory, or other mechanistic biomarkers. Consequently, the proposed differences in anti-inflammatory, anti-fibrotic, and intraglomerular hemodynamic effects between empagliflozin and canagliflozin could not be directly evaluated in this cohort. Prospective studies with larger populations, more comprehensive assessment of concomitant medications and potential confounders, standardized follow-up measurements, and clinically meaningful renal outcomes are warranted to further clarify the comparative renal effects of these two SGLT2 inhibitors.

## 5. Conclusions

In patients with type 2 diabetes mellitus without documented heart failure or ischemic heart disease, empagliflozin and canagliflozin demonstrated comparable longitudinal eGFR and UACR trajectories over 18 months. Although empagliflozin was associated with a higher likelihood of achieving a ≥30% reduction in UACR at 12 months, this difference was not sustained at 18 months. Furthermore, subgroup interaction analyses did not demonstrate significant effect modification by age or baseline albuminuria. Overall, our findings do not establish the superiority of either SGLT2 inhibitor in terms of renal outcomes. These results should be interpreted cautiously given the retrospective study design, baseline differences in renal function, incomplete follow-up measurements, and potential residual confounding. Further prospective studies with larger populations and comprehensive adjustment for confounding are warranted to clarify the comparative renal effects of these two SGLT2 inhibitors.

## Figures and Tables

**Table 1:** Baseline characteristics of patients treated with empagliflozin or canagliflozin.

Characteristic	Empagliflozin (*n* = 115)	Canagliflozin (*n* = 100)	*p* Value
Age, years, median (IQR)	63.1 (55.4–70.9)	62.8 (56.0–68.6)	0.593
Age group, years			0.285
<65	63 (54.8%)	62 (62.0%)	
≥65	52 (45.2%)	38 (38.0%)	
Sex			0.350
Female	39 (33.9%)	28 (28.0%)	
Male	76 (66.1%)	72 (72.0%)	
Body mass index, kg/m^2^, median (IQR)	26.5 (23.8–30.1)	26.9 (24.0–29.4)	0.794
Chronic kidney disease stage			0.180
Stage 1	31 (27.0%)	21 (21.0%)	
Stage 2	48 (41.7%)	39 (39.0%)	
Stage 3a	25 (21.7%)	19 (19.0%)	
Stage 3b	10 (8.7%)	17 (17.0%)	
Stage 4	1 (0.9%)	4 (4.0%)	
Glycated hemoglobin, %, median (IQR)	7.9 (7.1–8.7)	8.1 (7.1–9.2)	0.571
Serum creatinine, mg/dL, median (IQR)	0.9 (0.8–1.2)	1.1 (0.8–1.4)	0.016 *
Urinary albumin-to-creatinine ratio, mg/g, median (IQR)	26.0 (8.5–127.3)	35.3 (10.2–308.2)	0.403
Estimated glomerular filtration rate, mL/min/1.73 m^2^, median (IQR)	72.5 (56.3–93.2)	65.0 (47.9–85.7)	0.048 *
Diabetes kidney disease, *n* (%)	115 (100.0%)	100 (100.0%)	—
Hypertension, *n* (%)	66 (57.4%)	62 (62.0%)	0.492
Statin use, *n* (%)	90 (78.3%)	70 (70.0%)	0.166
Renin–angiotensin system inhibitor use, *n* (%)	59 (51.3%)	54 (54.0%)	0.693

Values are presented as median (interquartile range [IQR]) or number (percentage), as appropriate. *p* values were calculated using the Mann–Whitney U test for continuous variables and the chi-square test for categorical variables. * *p* < 0.05. Abbreviations: IQR, interquartile range.

**Table 2:** Longitudinal changes in UACR and eGFR in patients treated with empagliflozin or canagliflozin.

Time Point	Empagliflozin	Canagliflozin
Urinary albumin-to-creatinine ratio (UACR), mg/g		
Number of patients included in the complete-case longitudinal UACR analysis	*n* = 93	*n* = 80
Baseline	41.90 (9.40–149.60)	46.35 (12.20–493.83)
3 months	33.70 (10.55–140.30)	40.05 (12.05–423.20)
6 months	27.40 (8.90–134.20)	30.60 (12.80–204.82)
12 months	21.50 (8.38–139.45)	43.20 (17.38–307.65)
15 months	21.10 (9.58–138.50)	45.10 (14.00–233.95)
18 months	21.10 (10.80–106.05)	31.50 (12.40–210.13)
Within-group *p* value	0.001	0.449
Estimated glomerular filtration rate (eGFR), mL/min/1.73 m^2^		
Number of patients	*n* = 115	*n* = 100
Baseline	72.45 (56.26–93.17)	64.99 (47.87–85.66)
3 months	72.65 (52.96–93.69)	63.75 (46.87–85.80)
6 months	72.77 (52.91–94.79)	63.39 (46.02–86.26)
9 months	70.59 (54.60–92.02)	64.03 (45.25–82.11)
12 months	71.94 (54.60–92.02)	61.66 (44.95–82.14)
15 months	70.25 (54.12–89.75)	61.38 (43.46–80.48)
18 months	70.14 (50.35–88.35)	63.32 (41.48–81.08)
Within-group *p* value	<0.001	<0.001

Values are presented as median (interquartile range). Within-group changes over time were assessed using the Friedman test, followed by post hoc pairwise comparisons using the Dunn–Bonferroni method. Significant post hoc comparisons (*p* < 0.05) were as follows: in the empagliflozin group, UACR at 3 vs. 12 months and 3 vs. 18 months, and eGFR at baseline vs. 15 months, baseline vs. 18 months, and 3 vs. 18 months; in the canagliflozin group, eGFR at baseline vs. 12, 15, and 18 months. For the longitudinal UACR analysis, only patients with available UACR measurements at all scheduled assessment time points were included. Accordingly, the numbers of patients included in the UACR analysis and their baseline UACR values may differ from those reported in Table 1. This complete-case analysis was distinct from the time-specific paired analyses presented in Table 3, which included patients with both baseline and the corresponding follow-up UACR measurements. Abbreviations: eGFR, estimated glomerular filtration rate; UACR, urinary albumin-to-creatinine ratio.

**Table 3:** Comparison of changes in UACR and eGFR between empagliflozin and canagliflozin.

Outcome/Time Interval	*n*	Empagliflozin, Median (IQR)	*n*	Canagliflozin, Median (IQR)	*p* Value
HbA1c (18 months—baseline)	115	−0.50 (−1.70, 0.20)	100	−0.70 (−1.70, 0.30)	0.625
UACR (mg/g)					
3 months—baseline	106	0.00 (−12.36, 3.55)	90	0.00 (−13.78, 2.50)	0.723
6 months—baseline	99	−2.90 (−37.80, 4.80)	87	0.00 (−38.80, 2.90)	0.748
12 months—baseline	101	−2.90 (−36.32, 5.40)	88	−1.90 (−37.53, 10.08)	0.475
15 months—baseline	103	−1.75 (−51.30, 4.60)	88	−0.95 (−44.48, 19.98)	0.543
18 months—baseline	112	−1.60 (−49.20, 6.40)	98	0.35 (−33.78, 11.25)	0.312
eGFR (mL/min/1.73 m^2^)					
3 months—baseline	115	0.00 (−5.78, 2.71)	100	−1.60 (−5.34, 2.20)	0.531
6 months—baseline	115	−1.83 (−6.98, 3.64)	100	−1.85 (−6.22, 1.52)	0.538
9 months—baseline	115	−2.55 (−5.80, 3.07)	100	−2.78 (−6.81, 2.78)	0.345
12 months—baseline	115	−2.59 (−6.50, 3.07)	100	−2.74 (−8.89, 2.61)	0.353
15 months—baseline	115	−2.28 (−9.10, 4.04)	100	−3.42 (−10.34, 2.16)	0.264
18 months—baseline	115	−3.67 (−10.48, 4.03)	100	−4.09 (−10.10, 2.15)	0.789

Footnote: Values are median (interquartile range, IQR). Between-group comparisons were performed using the Mann–Whitney U test. For UACR analyses, changes from baseline were calculated only in patients with both baseline and the corresponding follow-up measurements available. Each follow-up time point was analyzed separately; therefore, the sample size varied across assessment intervals. Patients were not required to have measurements available at other follow-up time points. Missing measurements were not imputed.

**Table 4:** Adjusted Regression and Longitudinal Analyses of Renal Outcomes According to SGLT2 Inhibitor Treatment.

Outcome	Analysis	Follow-Up	Adjusted Estimate/*p* for Interaction	95% CI	*p* Value	*n*
Change in eGFR (ΔeGFR)	Linear regression	18 months	β = −0.432	−4.124 to 3.259	0.819	215
eGFR trajectory	Mixed-effects model	3–18 months	—	—	0.356	215
UACR reduction ≥30%	Logistic regression	12 months	OR = 0.398	0.201–0.790	0.008	189
UACR reduction ≥30%	Logistic regression	18 months	OR = 0.620	0.324–1.184	0.147	209
Log-UACR trajectory	Mixed-effects model	3–18 months	—	—	0.104	214

Adjusted estimates represent comparisons of canagliflozin with empagliflozin (reference group). The eGFR linear regression model was adjusted for baseline eGFR, age, sex, baseline HbA1c, hypertension, and baseline ACE inhibitor/ARB use; HC3 robust standard errors were applied. The UACR logistic regression models were adjusted for age, sex, baseline eGFR, baseline log-transformed UACR, HbA1c, hypertension, ACE inhibitor/ARB use, and statin use. A ≥30% reduction in UACR was defined as a follow-up UACR value ≤ 70% of baseline. The linear mixed-effects models included treatment group, follow-up time, and the treatment-by-time interaction as fixed effects, with patient-specific random intercepts. The eGFR model was adjusted for baseline eGFR, age, sex, baseline HbA1c, hypertension, and baseline ACE inhibitor/ARB use. The log-transformed UACR model was adjusted for baseline log-transformed UACR, baseline eGFR, age, sex, HbA1c, hypertension, ACE inhibitor/ARB use, and statin use. *p* values for mixed-effects models represent overall treatment-by-time interactions. ARB, angiotensin receptor blocker; CI, confidence interval; eGFR, estimated glomerular filtration rate; OR, odds ratio; UACR, urine albumin-to-creatinine ratio.

## Data Availability

The datasets generated and/or analysed during the current study are not publicly available due to the regulation of IRB in Taichung Veterans General Hospital but are available from the corresponding author on reasonable request.

## Supplementary Materials

The following supporting information can be downloaded at https://www.mdpi.com/article/10.3390/jcm15218631/s1. Supplementary Table S1. UACR and eGFR trends in empagliflozin and canagliflozin across different time periods in subgroups of young and elderly groups. Supplementary Table S2. Comparisons of UACR and eGFR (18 months compared to baseline) between empagliflozin and canagliflozin across different age and baselines. Supplementary Table S3. UACR and eGFR trends in empagliflozin and canagliflozin across different time periods in subgroups of baseline UACR < 200 mg/g and UACR ≥ 200 mg/g. Supplementary Table S4. Comparisons of UACR and eGFR (18 months compared to baseline) between empagliflozin and canagliflozin across different baseline UACR < 200 and UACR ≥ 200. Supplementary Table S5. Percentage change in UACR from baseline. Supplementary Table S6. Proportion of patients achieving a ≥30% reduction in UACR. Supplementary Figure S1. Patient selection algorithm.
