# Supplementary material for: Longitudinal Changes in Albuminuria and Estimated Glomerular Filtration Rate in Patients with Type 2 Diabetes Treated with Empagliflozin or Canagliflozin: A Real-World Cohort Study
Source: J Clin Med. 2026 Nov 5;15(21):8631. doi: 10.3390/jcm15218631 (PMC13763589; doi:10.3390/jcm15218631)

Supplementary data

Table S1. UACR and eGFR trends in empagliflozin and canagliflozin across different time periods in subgroups of young and elderly groups.

	Empagliflozin			Canagliflozin		
**UACR (mg/g)**						
Age<65	n=44			n=48		
Baseline	38.40	(8.35,	144.00)	48.05	(10.85,	1432.83)
3 months	32.95	(10.30,	132.94)	47.05	(10.45,	1203.83)
6 months	30.43	(8.56,	121.38)	39.54	(11.60,	907.63)
12 months	22.15	(7.18,	121.10)	43.90	(16.43,	1017.18)
15 months	17.75	(8.69,	129.93)	60.75	(15.28,	1002.90)
18 months	19.65	(9.95,	150.73)	31.50	(12.95,	734.50)
p value	0.163			0.424		
Age≥65	n=49			n=32		
Baseline	41.90	(10.30,	152.30)	36.65	(12.20,	125.58)
3 months	37.00	(10.75,	149.60)	32.95	(13.03,	135.30)
6 months	27.40	(9.40,	147.25)	25.95	(12.80,	122.31)
12 months	21.50	(9.45,	156.63)	43.20	(18.09,	116.69)
15 months	23.10	(11.75,	143.50)	43.40	(13.05,	90.15)
18 months	21.10	(12.85,	106.05)	33.75	(12.25,	98.68)
p value	**0.004****			0.957		
**eGFR** (ml/min/1.732m^2)						
Age<65	n=63			n=62		
Baseline	80.14	(66.24,	101.56)	73.59	(54.97,	90.57)
3 months	81.17	(64.59,	103.52)	71.21	(53.19,	91.11)
6 months	81.80	(63.26,	100.07)	73.54	(48.63,	91.88)
9 months	84.47	(60.31,	101.74)	72.83	(45.75,	85.28)
12 months	85.47	(61.87,	101.08)	70.73	(46.32,	87.55)
15 months	80.46	(62.47,	102.64)	68.32	(43.51,	84.57)
18 months	79.07	(58.66,	97.88)	70.12	(41.85,	85.59)
p value	**0.003****			**0.003****		
Age≥65	n=52			n=38		
Baseline	62.56	(50.39,	79.83)	53.31	(45.79,	73.71)
3 months	58.29	(45.77,	79.51)	50.74	(44.39,	73.93)
6 months	58.86	(44.90,	80.30)	52.75	(44.47,	70.25)
9 months	60.45	(48.03,	79.53)	51.14	(45.08,	70.17)

12 months	58.44	(48.52,	75.79)	54.36	(42.15,	69.41)
15 months	58.61	(47.07,	78.48)	51.21	(42.76,	73.73)
18 months	58.99	(44.07,	74.32)	51.33	(41.06,	73.86)
p value	0.054			0.073		

Friedman test. *p<0.05, **p<0.01

Post-hoc analysis (Dunn-Bonferroni) p<0.05

Empagliflozin

UACR(Age≥65) 0M vs 18M

eGFR(Age<65) 0M vs 18M

Canagliflozin

eGFR(Age<65) 0M vs 15M ;0M vs 18M

Table S2 Comparisons of UACR and eGFR (18 months compared to baseline) between empagliflozin and canagliflozin across different age and baseline.

	Empagliflozin(n=63)			Canagliflozin(n=62)			*p* value
**Age<65**							
UACR (mg/g)	-0.50	(-31.50,	6.78)	-0.30	(-123.18,	7.90)	0.719
eGFR (ml/min/1.732m^2)	-3.78	(-10.17,	4.03)	-4.33	(-12.28,	2.20)	0.463
HbA1c (%)	-0.40	(-1.50,	0.20)	-0.50	(-1.83,	0.40)	0.811
	Empagliflozin(n=52)			Canagliflozin(n=38)			
**Age≥65**							
UACR (mg/g)	-3.70	(-81.10,	6.40)	2.20	(-17.78,	23.13)	**0.043***
eGFR (ml/min/1.732m^2)	-3.22	(-12.02,	3.96)	-3.82	(-8.77,	1.87)	0.647
HbA1c (%)	-0.50	(-1.70,	0.25)	-0.75	(-1.45,	0.13)	0.635

Mann-Whitney U test. Median(IQR) *p<0.05, **p<0.01

Table S3. UACR and eGFR trends in empagliflozin and canagliflozin across different time periods in subgroups of baseline UACR<200 mg/g and UACR ≥200 mg/g

	Empagliflozin			Canagliflozin		
UACR (mg/g)						
Baseline UACR<200	n=76			n=54		
Baseline	18.50	(8.15,	88.28)	17.25	(7.23,	46.88)
3 months	21.10	(9.33,	73.78)	19.00	(7.80,	46.88)
6 months	15.83	(7.93,	74.63)	18.75	(10.00,	38.10)
12 months	15.70	(7.03,	66.25)	22.25	(11.28,	46.14)
15 months	16.25	(8.05,	63.63)	19.65	(11.53,	53.50)
18 months	17.20	(9.83,	44.05)	19.85	(9.38,	38.70)
p value	0.097			0.116		
Baseline UACR ≥200	n=17			n=26		
Baseline	1171.00	(688.45,	2217.50)	1365.55	(493.83,	2434.00)
3 months	1124.00	(510.55,	1761.50)	1203.55	(423.20,	2516.45)
6 months	916.00	(374.44,	1392.00)	899.61	(200.95,	2271.74)
12 months	708.00	(266.30,	1701.00)	960.65	(307.65,	2051.67)
15 months	594.00	(210.71,	2225.75)	967.74	(205.43,	1942.88)
18 months	726.00	(109.20,	2420.15)	797.50	(187.43,	1696.55)
p value	**0.003****			**0.045***		
eGFR						
Baseline UACR<200	n=97			n=72		
Baseline	76.05	(59.75,	95.40)	73.59	(53.42,	90.10)
3 months	75.66	(58.29,	96.27)	73.24	(50.92,	90.80)
6 months	76.54	(57.76,	96.85)	73.11	(52.40,	94.09)
9 months	75.70	(57.16,	95.08)	73.51	(51.67,	89.36)
12 months	75.70	(56.86,	94.69)	70.99	(54.74,	88.02)
15 months	75.27	(57.57,	98.88)	72.41	(53.32,	88.02)
18 months	72.70	(55.53,	95.25)	71.95	(51.16,	87.21)
p value	**0.040****			0.092		
Baseline UACR ≥200	n=18			n=28		
Baseline	56.74	(45.36,	66.01)	45.54	(35.65,	65.24)
3 months	53.49	(38.72,	68.03)	47.12	(36.79,	59.72)
6 months	52.40	(36.94,	60.90)	45.23	(36.03,	59.00)
9 months	52.40	(36.53,	59.08)	42.20	(36.42,	60.89)
12 months	53.76	(36.53,	63.48)	43.89	(34.60,	56.00)
15 months	49.49	(40.95,	65.33)	41.63	(31.67,	57.40)
18 months	48.63	(38.02,	57.48)	40.48	(27.95,	55.46)

p value	**<0.001****	**<0.001****

Friedman test. *p<0.05, **p<0.01

Post-hoc analysis (Dunn-Bonferroni) p<0.05

Empagliflozin

UACR(UACR≥200) 0M vs 15M; 0M vs 18M

eGFR(UACR≥200) 0M vs 9M;3M vs 18M

Canagliflozin

eGFR(UACR≥200) 0M vs 12M;0M vs 15M; 0M vs 18M;3M vs 18M

Table S4. Comparisons of UACR and eGFR (18 months compared to baseline) between empagliflozin and canagliflozin across different baseline UACR<200 and UACR ≥200

	Empagliflozin(n=97)			Canagliflozin(n=72)			*p* value
**UACR<200**							
UACR (mg/g)	-0.60	(-19.60,	6.90)	1.70	(-3.30,	11.10)	**0.023***
eGFR (ml/min/1.732m^2)	-2.25	(-10.29,	4.87)	-2.14	(-8.50,	3.39)	0.934
HbA1c (%)	-0.50	(-1.60,	0.10)	-0.70	(-1.40,	0.30)	0.820
	Empagliflozin(n=18)			Canagliflozin(n=28)			
**UACR≥200**							
UACR (mg/g)	-198.45	(-735.78,	182.50)	-304.70	(-758.00,	44.90)	0.711
eGFR (ml/min/1.732m^2)	-7.26	(-12.42,	-4.11)	-8.08	(-13.08,	0.17)	0.946
HbA1c (%)	0.00	(-1.73,	0.75)	-0.70	(-2.95,	0.07)	0.137

Mann-Whitney U test. Median(IQR) *p<0.05, **p<0.01

Table S5. Percentage change in UACR from baseline

Values are median (interquartile range, IQR) of individual patient-level percentage changes.

Follow-up	Empagliflozin, median % (IQR)	Canagliflozin, median % (IQR)	Between-group P value
3 months	0.0 (-24.4, 26.4); n=106	0.0 (-18.2, 10.7); n=90	0.764
6 months	-15.3 (-42.9, 35.9); n=99	0.0 (-33.8, 22.2); n=87	0.463
12 months	-19.7 (-49.7, 46.6); n=101	-3.2 (-31.4, 61.7); n=88	0.075
15 months	-8.5 (-47.6, 58.0); n=103	-3.2 (-41.3, 60.6); n=88	0.333
18 months	-10.4 (-52.5, 74.0); n=112	4.3 (-46.2, 68.8); n=98	0.361

Percentage change was calculated as [(follow-up UACR − baseline UACR) / baseline UACR] × 100. Negative values indicate a reduction in UACR. Only patients with available paired baseline and follow-up UACR measurements were included at each time point. Between-group comparisons were performed using the Mann–Whitney U test.

Table S6 Proportion of patients achieving a ≥30% reduction in UACR

Values are number of responders / number with paired measurements (%).

Follow-up	Empagliflozin, n/N (%)	Canagliflozin, n/N (%)	Between-group P value
3 months	25/106 (23.6%)	16/90 (17.8%)	0.380
6 months	32/99 (32.3%)	25/87 (28.7%)	0.635
12 months	45/101 (44.6%)	23/88 (26.1%)	0.010
15 months	45/103 (43.7%)	29/88 (33.0%)	0.139
18 months	44/112 (39.3%)	32/98 (32.7%)	0.388

A responder was defined as a ≥30% reduction in UACR from baseline, corresponding to follow-up UACR ≤70% of baseline UACR. Only patients with available paired baseline and follow-up UACR measurements were included at each time point. Between-group comparisons were performed using Fisher's exact test. P values are unadjusted for multiple comparisons.

Supplementary figure S1. Patient selection algorithm

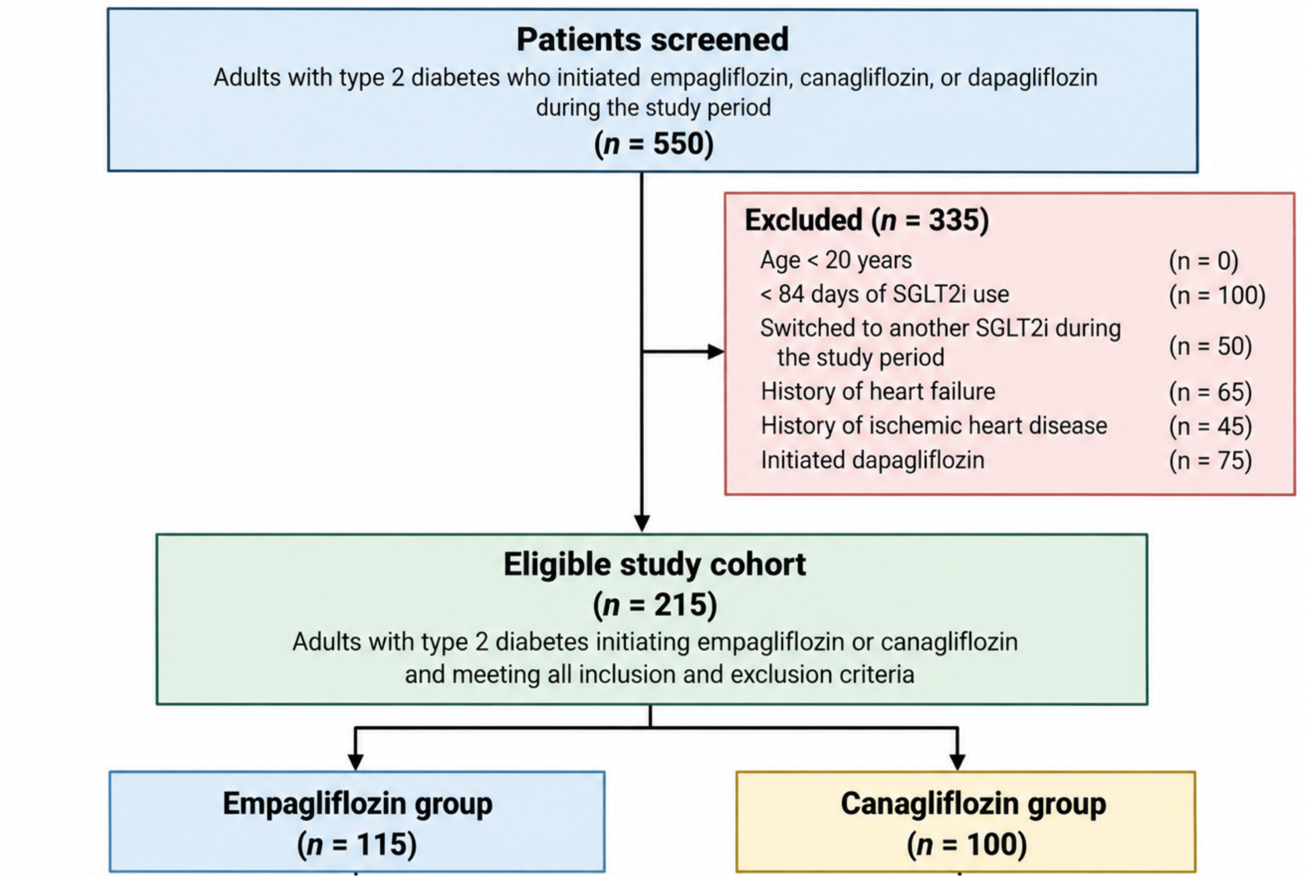

Supplement: Supplementary file 1 [file jcm-15-08631-s001.zip › jcm-4655844-supplementary.pdf]
